# Effect of Heeled Shoes on Joint Symptoms and Knee Osteoarthritis in Older Adults: A 5‐Year Follow‐Up Study

**DOI:** 10.1002/acr2.11298

**Published:** 2021-07-20

**Authors:** Thomas A. Perry, Charlotte Dando, Tim D. Spector, Deborah J. Hart, Catherine Bowen, Nigel Arden

**Affiliations:** ^1^ University of Oxford Oxford United Kingdom; ^2^ University of Southampton Southampton United Kingdom; ^3^ King's College London London United Kingdom; ^4^ University of Oxford, Oxford, United Kingdom, and University of Southampton Southampton United Kingdom

## Abstract

**Objective:**

Our aims were to examine the effects of heeled shoes on incident knee osteoarthritis (OA) and joint pain.

**Methods:**

We used longitudinal data from the Chingford 1000 Women Study (Chingford Study), a prospective cohort of women aged 50 years or older. Participants with musculoskeletal disorders and/or a history of knee‐related injury/surgery were excluded. Participants were followed for up to 5 years for incident outcomes including *1*) radiographic knee OA (RKOA) and *2*) joint pain (feet, knees, hips, and back). Footwear data, including ever worn heels of 2 inches or more and daytime/evening hours (per week) spent wearing heeled shoes over five decades (ages <20 years, 20‐30 years, 30‐40 years, and >50 years), were available at Year 10 whereas knee radiographs and joint symptom data were also collected at Year 15. Cumulative time spent wearing heeled shoes was calculated for women reporting ever‐use of heeled shoes (≥2 inches). Multiple logistic regression was used to examine the relationship between exposures and outcomes (from Year 10 to Year 15).

**Results:**

A total of 356 women were eligible at Year 10 with a median (interquartile range) age of 60 (56‐65) years. Compared with non‐use, ever‐use of heeled shoes (≥2 inches) was not associated with incident RKOA (1.35; 95% confidence interval: 0.56‐3.27). No associations were observed between increasing cumulative time spent wearing heels and incident outcomes.

**Conclusion:**

Compared with the non‐use of heeled shoes, ever‐use of heels (≥2 inches) was not associated with incident RKOA and incident joint symptoms. Further, increasing cumulative time spent wearing heels was not associated with any of our outcomes.

**Significance & Innovations**

- No associations were observed between two heel‐related exposures, ever‐use (yes/no) of heeled shoes (≥2 inches) and cumulative time spent wearing heeled shoes, and incident radiographic knee osteoarthritis.
- No relationship was observed between heel‐related exposures and incident knee, hip, and foot pain. It is unlikely that ever‐use of heeled shoes is associated with incident back pain.
- Further studies are required to examine the involvement and effect of heeled shoes on the ankle joint.

## INTRODUCTION

The effect of footwear as a modifiable factor for the prevention of joint symptoms and knee osteoarthritis (OA) appears to be important; however, there is insufficient and often conflicting evidence regarding its efficacy (1, 2). As part of routine clinical care, health professionals often attempt to modify footwear habits either as a primary intervention for lower‐limb (eg, foot and ankle) symptoms or as a preventative measure in the development of lower‐limb OA. Treatment recommendations for the nonsurgical management of knee OA are, however, not consistent with some (3, 4), but not all (5), physicians recommending “modified shoes” as either part of core treatment or as an intervention further along the treatment algorithm.

As well as being a potentially important intervention strategy, footwear is often implicated in joint pathology. Abnormal knee loading has been associated with increased risk of knee OA (6), knee torque (7) and, foot position and motion have been associated with knee load (8, 9, 10); in addition, footwear has significant effects on biomechanics (11). With a specific focus on heeled shoes, wearing heels can increase muscle activity (12) and increase knee flexion, plantar flexion, anterior pelvic tilt, and trunk extension (13). Together, these changes in kinematics can lead to adverse repetitive dynamic loading which leads to joint pain/OA in the lower and upper limbs. These effects can be seen most prominently in elderly individuals, with footwear linked to the development of OA within the foot (14), knee (10, 15), and hip (16). In particular, older women are identified as often wearing incorrectly fitting footwear and wearing heels higher than approximately 1‐inch which are associated with foot pathology (17), increased risk of falling (18), impairment in standing and leaning balance (19), and alterations in lower‐limb and back muscle activation (20).

Because of the relationship between footwear and joint pathology, footwear and heel height has been the subject of several investigations in relation to foot pain, with little attention on other joint sites, yet the evidence remains inconclusive. For instance, in a Brazil‐based study, 50% of women reported foot pain, but this was not associated with current high‐heeled shoe use (21). Previously, in the United Kingdom (UK), Dawson et al (14) reported that the prevalence of foot problems was 83% in a sample of 127 older women (aged 50‐70 years) who had worn shoes with 1‐inch heels regularly at some time. A surprising finding was that a number of foot problems were associated with wearing lower‐than‐average heels, which challenges the belief that wearing high heels is detrimental to foot health (14). Interestingly, although Dawson et al (14) reported that most older women had been exposed to high‐heeled shoes over many years, data from 3378 members of the Framingham study suggest that women who regularly wore high heels in the past were more likely to experience foot pain in their later years (22). Additional studies are required, including studies of the hips (23), to examine the effects of heeled shoes on incident joint symptoms not only of the feet but also on other biomechanically involved sites.

There is now an opportunity to retrospectively examine the associations between wearing heeled shoes and joint pain and OA using data from the Chingford 1000 Women Study (Chingford Study). It is anticipated that this may lead to a greater understanding of this relationship when considering strategies to prevent joint pain in rheumatology‐related foot health. Our aim was, therefore, to examine the effect of ever‐use of shoes with heels of 2 inches or more and, in those with positive responses, to examine the effect of life‐time cumulative wear on incident radiographic knee OA (RKOA) and incident joint pain in women aged 50 years or older.

## PATIENTS AND MATERIALS

### Study sample

This study was conducted retrospectively using data from the Chingford Study, a prospective, population‐based longitudinal cohort of 1003 women aged 45 to 64 years (mean: 54.2 years) (24). The women have been assessed annually and are representative of women from the UK population in terms of demographic characteristics (25).

For the current study, we used data acquired at Year 10 (9 years of follow‐up) and Year 15 (14 years of follow‐up). Participants were eligible for the primary analysis (ie, incident RKOA) if they had no evidence of RKOA (Kellgren and Lawrence [KL] grade ≥2 [26]) in both knees, were free of other musculoskeletal disease (eg, rheumatoid arthritis), and had no history of knee injury requiring 1‐week rest at Year 10. Further, participants with evidence of knee replacement at Year 10 and those who had a knee replacement during follow‐up were excluded. For the incident joint symptoms analysis, participants were eligible if they had no joint pain at the respective site, were free of musculoskeletal disease (eg, rheumatoid arthritis), and had no history of fracture at the respective site in the last 12 months. Knee‐related fractures included fractures at the femur, tibia, and fibula; foot‐related fractures included fractures at the toe, metatarsal bones, and metatarsophalangeal bones; and back‐related fractures included fractures at the vertebrae, ribs, and clavicle. There were no reports of hip‐related fractures in our study sample.

Weight‐bearing anteroposterior‐view radiographs of the knees (left/right) for all participants were acquired at Years 10 and 15. Knee radiographs were graded on a 0 to 4 scale across the whole knee joint using KL criteria (26). Radiographic OA of the whole knee was defined as a KL score of 2 or more (at the person level).

At Year 10 and Year 15, participants were asked joint‐specific and, when appropriate, side‐specific (left/right) joint pain questions for the knees, hips, and feet. In addition, symptom questions for the back, comprising upper and lower regions, were also asked. Joint symptoms were assessed using the National Health and Nutrition Examination Survey (27). Specifically, participants were asked to report “any episodes of pain in past year.” Women who responded positively were asked to report the number of “days with pain in the last month,” with categories including *1*) 0 days, *2*) 1 to 5 days, *3*) 6 to 14 days, and *4*) >15 days. Participants were classified at Year 10 as having current joint pain if the duration of pain was more than 15 days in the past month. Women who reported a value for days of pain in the past month but had a missing or zero entry for “any episodes of pain in past year” were also included in the analysis; there were few occurrences of this. Pain for most days in the previous month has been shown to be the most appropriate outcome/exposure for the investigation of symptom development in OA research (28).

### Predictor variable

At Year 10, all women were asked to complete a nurse‐administered standardized questionnaire that included questions related to footwear. Specifically, participants were asked whether they had “ever worn shoes with heels 2 inches high or more.” Women who responded positively to this question were then asked to provide information on *1*) the “heel height worn” (continuous), *2*) the “number of daytime hours per week of wearing” and *3*) the “number of evening hours per week of wearing” for each of the following age groups: less than 20 years, 20 to 30 years, 30 to 40 years, 40 to 50 years, and more than 50 years. Women who had a negative response to ever‐use were not required to complete these fields. For women who responded positively to whether they had “ever worn shoes with heels 2 inches high or more” and had a missing value for “heel height worn” for a given decade, their missing responses for daytime and evening wear were assumed to be missing because of non‐use in the given decade. All women who had a positive entry for ever‐use and had missing entries for heel height at a given decade also had missing data on daytime and evening wear. We calculated the cumulative time spent wearing heeled shoes through summing daytime and evening hours across the five decades. Our two exposures included *1*) ever worn heels of 2 inches or more (yes/no) and, in participants having worn heels, *2*) cumulative time spent wearing heels across five decades.

### Outcome variables

#### Incident RKOA

In those with no evidence of RKOA at baseline (Year 10) (KL grade <2 in both knees), incident RKOA was defined as the occurrence of a KL grade of 2 or more in either/both knees during follow‐up.

#### Incident joint symptoms

Incident joint symptoms were assessed for the knees, feet, hips, and back. In participants with no evidence of joint pain at the site investigated at Year 10, incident joint symptoms were defined as the occurrence of pain at follow‐up (Year 15) (person‐level). Participants were classified as having current pain if they reported pain for most days in the previous month, in accordance with previous guidelines (28).

### Statistical analysis

Descriptive statistics were tabulated with normally distributed variables presented as means and standard deviations (SDs) and non‐normally distributed variables presented as medians and interquartile ranges (IQRs). Categorical variables were presented as counts and percentages. Data were analyzed using STATA version 15.1 (StataCorp). To examine the relationship between our two heel‐related exposures (ie, ever‐use [yes/no] and cumulative time spent wearing heels [continuous]) and incident outcomes, we used logistic regression modelling with *1*) incident RKOA and *2*) incident joint symptoms, (eg, knee, foot, hip, and back) as the respective outcomes. Results were presented as odds ratios (ORs) with 95% confidence intervals (CIs) for crude and adjusted models. We simultaneously controlled for potential confounders using multiple logistic regression. Cumulative time spent wearing heels (hours) across five decades was categorized into quartiles because of the suspected nonlinear effects on the outcomes.

### Assessment of covariates

Covariates that were adjusted for included age and body mass index (BMI), which was calculated as weight (in kg)/height (in m^2^), as measured at Year 10. Additional covariates included previous occupation (measured at Year 1); participants were asked to report their previous occupational job category (eg, farming) (see supplementary material). Categories were assigned to levels of workload (sedentary, light, light manual, and heavy manual) in accordance with published methods (29). For the incident joint symptoms analyses, we further adjusted for baseline RKOA severity (ie, KL score in left and right knees) and knee symptom status (person‐level), as there is evidence to suggest that knee pain increases the risk of developing pain at sites outside the knee (30). We did not adjust for previous injury, as this is likely on the causal pathway.

## RESULTS

**Figure 1:**
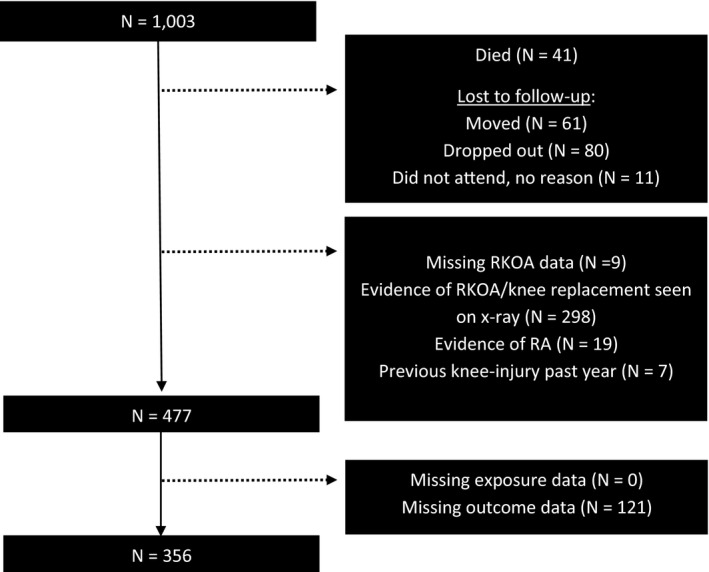
Flow chart of eligible study participants for primary analysis (incident radiographic knee osteoarthritis [RKOA]). RA, rheumatoid arthritis.

Of 1003 study participants, 356 women were eligible and had heel‐related footwear data at Year 10 (see Figure 1).

Characteristics of the eligible study sample for the primary analysis (incident RKOA) are shown in Table 1. The median age was 60 years (range: 56 to 65 years) and mean BMI was 25.8 (SD: 3.9). A total of 306 (86.0%) women had positive responses to ever having worn shoes with heels of 2 inches or more; of the sites assessed, back pain was the most common type of joint pain occurring in 18.0% of participants. Of the 306 women reporting having ever worn heels of 2 inches or more, 101 (33.0%) reported wearing heels across all five decades whereas 28 (9.2%) women reported wearing heels for one decade only. Further, the mostworn heel type between the ages of less than 20 years and 30 years was a stiletto and the mostworn heel type between the ages of 30 years and more than 50 years was a court shoe.

**Table 1:** Subject characteristics of eligible study sample for primary analysis at Year 10 (incident radiographic knee OA)

Characteristics (N = 356)	Results
Age, years, median (IQR)	60 (56‐65)
BMI, kg/m^2^, mean (SD)	25.8 (3.9)
Joint pain (yes), n (%)^a^	
Knee	33 (9.3)
Missing	2 (0.6)
Foot	30 (8.4)
Missing	0 (0)
Hip	35 (9.8)
Missing	4 (1.1)
Back	64 (18.0)
Missing	1 (0.3)
Ever worn shoes with heels ≥2 inches, n (%)	
No	50 (14.0)
Yes	306 (86.0)
In participants with positive responses for ever worn heels ≥2 inches	
Heels worn across the decades, yes, n (%)	
<20 years	270 (88.2)
20‐30 years	279 (91.2)
30‐40 years	220 (71.9)
40‐50 years	160 (52.3)
>50 years	118 (38.6)
Heel height worn across the decades, mean (SD)	
<20 years	2.82 (0.68)
20‐30 years	2.64 (0.62)
30‐40 years	2.38 (0.55)
40‐50 years	2.32 (0.51)
>50 years	2.26 (0.44)
Cumulative time spent wearing heels, hours/week, median (IQR)	
Daytime	70 (35‐135)
Evening	28 (12‐46)
Total	105 (48‐176)

Abbreviation: BMI, body mass index; IQR, interquartile range; OA, osteoarthritis

^a^ In either or both joint(s) (eg, left/right knee[s]).

A single study participant reported “no” to having ever worn heeled shoes (≥2 inches) but had data on heel height and daytime/evening wear across the decades, and she was subsequently included in all analyses. Further, an additional two participants had data on daytime/evening wear but had missing data for heel height at a particular decade; these participants were also included in all analyses. Further, in the fifth decade (>50 years), one participant reported heel height as 2 to 4 on a continuous scale (inches), and this was relabelled as 3 inches. All study participants with positive responses to having ever worn heeled shoes (≥2 inches) reported at least 1 hour per week of wearing time during at least one of the five decades.

In participants with no evidence of RKOA, women reporting ever‐use of heeled shoes (≥2 inches) was not statistically significantly associated with incident knee OA compared with non‐use in multivariate analysis (1.35; 95% CI: 0.56‐3.27) (Table 2). In women reporting ever‐use of heeled shoes, increasing cumulative time spent wearing heels was not statistically significantly associated with incident RKOA in the multivariate analysis.

**Table 2:** Association between heel exposures and incident RKOA

Exposure^a^	Univariate	*P* Value	Multivariate^b^	*P* Value
Ever worn heels ≥2 inches^c^	N = 356	‐	N =336	‐
No (n = 50 and n = 7)	1 (reference)	‐	1 (reference)	‐
Yes (n = 306 and n =59)	1.47 (0.63‐3.43)	0.38	1.35 (0.56‐3.27)	0.51
Cumulative wearing time^c^	N = 306		N = 288	
Second quartile (n = 87 and n = 13)	0.63 (0.23‐1.75)	0.37	0.53 (0.18‐1.56)	0.25
Third quartile (n = 91 and n = 19)	0.94 (0.35‐2.51)	0.91	0.98 (0.36‐2.67)	0.96
Fourth quartile (n = 96 and n = 20)	0.94 (0.36‐2.49)	0.90	0.95 (0.35‐2.57)	0.91

Abbreviation: RKOA, radiographic knee osteoarthritis.

All results are presented as odds ratios with 95% confidence intervals. Reference groups are indicated within the table.

^a^ All comparisons are made against those of the lowest quartile of cumulative time spent wearing heels (hours per week).

^b^ Adjusted for age, body mass index, and previous occupation.

^c^ Number of participants per strata with the number of incident case(s) per strata.

Compared with women reporting non‐use of shoes with heels of 2 inches or more, ever‐use was not associated with incident knee pain (0.96; 95% CI: 0.49‐1.92), foot pain (0.80; 95% CI 0.37‐1.73), or hip pain (1.61; 95% CI: 0.64‐4.05) (Table 3). However, in the multivariate analysis, the relationship between the ever‐‐use of shoes with heels 2 inches or more and incident back pain was of borderline statistical significance, in a protective direction, based on conventional standards (0.49; 95% CI: 0.24‐1.00).

**Table 3:** Association between heel exposure (ever worn heels ≥2 inches, yes/no) and incident joint symptoms

Exposures^a^	Univariate	*P* Value	Multivariate	*P* Value
*Ever Worn Heels (≥2 inches)*				
*Knee pain*	N = 512		N = 483	
*No (n = 78 and n = 13)*	1 (reference)	‐	1 (reference)	‐
*Yes (n = 434 and n = 77)*	1.08 (0.57‐2.05)	0.82	0.96 (0.49‐1.92)^b^, ^c^	0.92
*Foot pain*	N = 550		N = 519	
*No (n = 85 and n = 10)*	1 (reference)	‐	1 (reference)	‐
*Yes (n = 465 and n = 45)*	0.80 (0.39‐1.66)	0.56	0.80 (0.37‐1.73)^b^, ^d^	0.56
*Hip pain*	N = 540		N = 506	
*No (n = 82 and n = 8)*	1 (reference)	‐	1 (reference)	‐
*Yes (n = 458 and n = 48)*	1.08 (0.49‐2.38)	0.84	1.61 (0.64‐4.05)^b^, ^d^	0.31
*Back pain*	N = 482		N = 451	
*No (n = 71 and n = 17)*	1 (reference)	‐	1 (reference)	‐
*Yes (n = 411 and n = 50)*	**0.44 (0.24‐0.82)**	**0.01**	**0.49 (0.24‐1.00)** ^b^, ^d^	**0.05**

All results are presented as odds ratios with 95% confidence intervals. Reference groups are indicated within the table.

Statistically significant results are shown in bold.

^a^ Number of participants per strata and the number of incident case(s) per strata.

^b^ Adjusted for age, body mass index, and previous occupation.

^c^ Adjusted for radiographic knee osteoarthritis severity (as Kellgren and Lawrence scores for left and right knees).

^d^ Adjusted for radiographic knee osteoarthritis severity (as Kellgren and Lawrence scores for left and right knees) and knee pain status (pain for most days in the past month, yes/no).

Similar to the univariate analysis, after adjusting for age, BMI, previous occupation, and RKOA severity, there was no statistically significant association between cumulative time spent wearing heels and incident knee pain (Table 4). Specifically, compared with the lowest quartile, no association was observed for the second (0.61; 95% CI: 0.26‐1.48), third (0.96; 95% CI: 0.41‐2.25), and fourth quartiles (0.41; 95% CI: 0.16‐1.04). Similarly, no associations were observed between increasing quartiles and incident foot, hip, and back pain.

**Table 4:** Association between heel exposure (cumulative time spent wearing heels) and incident joint symptoms

Exposure^a^	Univariate	*P* Value	Multivariate^a^	*P* Value
*Cumulative Time Spent Wearing Heels*				
*Knee pain*	N = 434		N = 408	
*Second quartile (*n *= 132* and n = *23)*	0.73 (0.32‐1.64)	0.44	0.61 (0.26‐1.48)^b^, ^c^	0.28
*Third quartile (*n *= 125* and n = *28)*	1.00 (0.45‐2.20)	0.99	0.96 (0.41‐2.25)^b^, ^c^	0.93
*Fourth quartile (*n *= 128* and n = *15)*	0.46 (0.19‐1.08)	0.08	0.41 (0.16‐1.04)^b^, ^c^	0.06
*Foot pain*	N = 465		N = 437	
*Second quartile (*n *= 139* and n = *15)*	1.42 (0.45‐4.50)	0.55	1.06 (0.32‐3.54)^b^, ^d^	0.92
*Third quartile (*n *= 139* and n = *14)*	1.32 (0.41‐4.20)	0.64	1.18 (0.36‐3.92)^b^, ^d^	0.78
*Fourth quartile (*n *= 136* and n = *12)*	1.14 (0.35‐3.70)	0.83	1.05 (0.31‐3.53)^b^, ^d^	0.93
*Hip pain*	N = 458		N = 427	
*Second quartile (*n *= 137* and n = *17)*	3.4 (0.76‐15.28)	0.11	3.06 (0.66‐13.91)^b^, ^d^	0.16
*Third quartile (*n *= 143* and n = *17)*	3.24 (0.72‐14.55)	0.13	3.17 (0.69‐14.54)^b^, ^d^	0.14
*Fourth quartile (*n *= 128* and n = *12)*	2.48 (0.54‐11.52)	0.25	2.55 (0.54‐12.02)^b^, ^d^	0.24
*Back pain*	N = 411		N = 384	
*Second quartile (*n *= 123* and n = *18)*	1.17 (0.43‐3.16)	0.76	1.00 (0.35‐2.84)^b^, ^d^	0.99
*Third quartile (*n *= 127* and n = *17)*	1.06 (0.39‐2.86)	0.92	0.99 (0.35‐2.77)^b^, ^d^	0.98
*Fourth quartile (*n *= 114* and n = *9)*	0.59 (0.20‐1.75)	0.34	0.49 (0.16‐1.56)^b^, ^d^	0.23

All results are presented as odds ratios with 95% confidence intervals. All comparisons are made against those of the lowest quartile (quartile 1) of cumulative time spent wearing heels (hours/week).

^a^ Number of participants per strata and number of incident case(s) per strata.

^b^ Adjusted for age, body mass index, and previous occupation.

^c^ Adjusted for radiographic knee osteoarthritis severity (as Kellgren and Lawrence scores for left and right knees).

^d^ Adjusted for radiographic knee osteoarthritis severity (as Kellgren and Lawrence scores for left and right knees) and knee pain status (pain for most days in the past month, yes/no).

## DISCUSSION

We examined whether ever‐use of shoes with heels of 2 inches or more and cumulative time spent wearing heeled shoes were associated with incident RKOA and joint symptoms. No relationship was observed between both of our heel‐related exposures and incident RKOA and incident knee, hip, and foot pain, respectively. A statistically significant negative relationship was observed between ever‐use of shoes with heels of 2 inches or more and incident back pain although this was likely a consequence of unmeasured, residual confounding and type 1 error.

Wearing of high‐heeled shoes has been shown to increase forces across the patellofemoral joint, lead to greater compressive force at the medial compartment, and increase knee flexion and varus moments (10, 31) which may predispose individuals to later degenerative joint changes. However, there are few data describing the relationship between high heels and knee OA, and previous studies investigating the effects of high heels on the risk of knee OA have revealed varying results (2, 10, 31, 32, 33). Consequently, the clinical message regarding the use of such footwear is unclear. Typically, lower‐extremity muscle strengthening is recommended to help decrease knee loading in high‐heel users.

In a recent systematic review and meta‐analysis, Nguyen et al reported that, of 203 participants across 14 studies, high heels were associated with increased knee flexion moment, flexion angle, and varus moment; thus, the authors concluded that high heels likely increase susceptibility to knee OA (31). This review, however, highlighted a need for prospective evaluation and the use of large observational data to examine this relationship. In a separate case‐control study, McWilliams et al reported that the persistent use of high‐heeled shoes between the ages of 21 years and 50 years was associated with a decreased risk of knee OA in univariate analysis (OR: 0.55; 95% CI: 0.33‐0.90), although, after adjustment for age, BMI, occupation, and previous injury, no association was observed (34). Our study goes beyond this to examine the relationship between two heel‐related exposures (ie, ever‐use (yes/no) and cumulative time spent wearing heels) and incident knee OA using a large prospective dataset.

After adjustment for potential confounders, no association between the ever‐use of shoes with heels of 2 inches or more and cumulative time spent wearing heeled shoes and incident knee OA was observed. This is in agreement with previous data. For instance, using a case‐control design of 111 women waiting for knee replacement, Dawson et al reported that the ever‐use of high‐heeled shoes (2‐3 inches) was associated with a reduced risk of symptomatic knee OA, although this finding was not statistically significant (32). Similarly, in a separate case‐control study, McWilliams et al reported that the persistent use of heels during early adulthood was not associated with the risk of knee OA, though in univariate analysis, the findings pointed towards a negative association between persistent use of high/narrow women’s heels and lower‐limb OA (33). A possible explanation for a negative relationship between regular use of heeled shoes and the risk of knee OA could be that these women are from a reduced risk group (ie, have a lower BMI and/or are exposed to fewer occupational risk factors [eg, lifting/carrying goods]).

In the current study, we observed a statistically significant negative association between ever‐use of shoes with heels of 2 inches or more and incident back pain after adjustment for participant demographics and other structural/symptom endpoints. The extent to which a participant’s posture and spinal position is modified by shoes with heels during dynamic activities is highly contested. There is evidence to suggest that lumbar lordosis, an exaggerated inward curve of the spine, is associated with lower back pain (35), and shoe heels may (36) or may not (37, 38) decrease lumbar lordosis. Further, there is conflicting evidence on whether shoe heels affect pelvis and trunk movement during gait within different female populations (39, 40, 41). Although there is an acknowledgment that compensation occurs during gait, this is unique for each individual when wearing heels at different heights (39, 41), and whether this places a person at higher risk of developing future complications is unknown. Coupling this with the natural ageing process in which it is known that balance declines and changes in gait occur, of which these can affect a person's stability, muscular strength, and functional mobility (42), it would be hard to determine the true association of heel height and back pain from this data alone. Back pain encompasses physical, emotional, and psychological risk factors (43, 44). In our analysis, we undertook several comparisons, and it is possible that this observed finding may be due to type 1 error. When examining the relationship between cumulative time spent wearing heels and incident back pain, no relationship was observed (Table 4). Given that no such association was observed when using the more sensitive exposure of cumulative time spent wearing heels, we are confident that the use of heeled shoes (≥2 inches) is unlikely to be associated with incident back pain. Further studies are, however, needed to confirm these findings.

There are many strengths to this study. We used data from a large observational dataset of women that had well‐characterized data on footwear. To our knowledge, this is the first study to examine the effect of two heel‐related exposures on incident RKOA and incident joint pain.

There are several potential limitations to this study. Firstly, we were unable to examine the presence of hip, foot, or spinal OA at Year 10 as X‐rays for such joints/regions were not acquired as part of the study protocol. Subsequently, when examining the relationship between heel exposures and incident joint pain, we were unable to control for OA status, except for RKOA status. An additional limitation was that we were unable to account for heel wear during the ages of 50 years to 60 years or more. Median age at Year 10 was 60 years (range: 56 to 65 years) and we had data for the ages of 20 years to more than 50 years. Subsequently, we were unable to account for possible heel use during the early years of participation in the Chingford Study. This, however, is unlikely to affect our findings, as the average time spent wearing heels decreased with age. More so, we were unable to assess the validity of the shoe‐related predictors. It is likely that our findings were subject to recall bias, as the exposures were recalled over five decades. Further work is required to confirm our findings. In addition, future studies should aim to examine the relationship between changes in footwear and risk of knee OA and joint symptom development. Although we adjusted for previous occupational status, there is likely to be residual confounding, as age at the start of occupation and occupational duration were not reported. Therefore, we cannot confirm whether adjustment for previous occupational status overlapped with the decades of 20 years to more than 50 years. Also, the absence of previous injury data, beyond joint fracture, and knee surgery meant that these variables could not be adjusted for within our analyses. Therefore, we cannot exclude the potential effects of such factors on our models. Lastly, we did not include women who had a total knee replacement during follow‐up as incident knee OA cases.

In conclusion, compared with women who had never worn heeled shoes (≥2 inches), the use of heeled shoes was not associated with incident RKOA and incident knee, hip, and foot joint symptoms. Although a statistically significant relationship was observed between ever‐use of heeled shoes and incident back pain, this was likely a result of type 1 error and unmeasured residual confounding. Lastly, in those reporting having worn heeled shoes, increasing time spent wearing heels was not associated with any of our outcomes. These data challenge the belief that wearing heeled shoes is detrimental to foot health, though further study is required to confirm the involvement and effects of heeled shoes on the ankle joint.

## AUTHOR CONTRIBUTIONS

All authors were involved in drafting the article or revising it critically for important intellectual content, and all authors approved the final version to be published. Dr. Perry had full access to all of the data in the study and takes responsibility for the integrity of the data and the accuracy of the data analysis.

### Conception and Design

Perry, Dando, Bowen.

### Acquisition of data

Spector, Hart.

### Analysis and interpretation of data

Perry, Dando, Bowen, Arden.

## Supporting information

Table S1

## References

[1] Wagner A , Luna S . Effect of footwear on joint pain and function in older adults with lower extremity osteoarthritis. J Geriatr Phys Ther 2018;41:85–101. 27824657 10.1519/JPT.0000000000000108

[2] Barnish MS , Barnish J . High‐heeled shoes and musculoskeletal injuries: a narrative systematic review. BMJ Open 2016;6:e010053. 10.1136/bmjopen-2015-010053 PMC4735171 26769789

[3] Zhang W , Moskowitz RW , Nuki G , Abramson S , Altman RD , Arden N , et al. OARSI recommendations for the management of hip and knee osteoarthritis, part II: OARSI evidence‐based, expert consensus guidelines. Osteoarthritis Cartilage 2008;16:137–62. 18279766 10.1016/j.joca.2007.12.013

[4] Fernandes L , Hagen KB , Bijlsma JW , Andreassen O , Christensen P , Conaghan PG , et al. EULAR recommendations for the non‐pharmacological core management of hip and knee osteoarthritis. Ann Rheum Dis 2013;72:1125–35. 23595142 10.1136/annrheumdis-2012-202745

[5] Kolasinski SL , Neogi T , Hochberg MC , Oatis C , Guyatt G , Block J , et al. 2019 American College of Rheumatology/Arthritis Foundation guideline for the management of osteoarthritis of the hand, hip, and knee. Arthritis Care Res 2020;72:149–62. 10.1002/acr.24131 PMC11488261 31908149

[6] Maly MR . Abnormal and cumulative loading in knee osteoarthritis. Curr Opin Rheumatol 2008;20:547–52. 18698176 10.1097/BOR.0b013e328307f58c

[7] Kerrigan DC , Johansson JL , Bryant MG , Boxer JA , Della Croce U , Riley PO . Moderate‐heeled shoes and knee joint torques relevant to the development and progression of knee osteoarthritis. Arch Phys Med Rehabil 2005;86:871–5. 15895330 10.1016/j.apmr.2004.09.018

[8] Levinger P , Menz HB , Morrow AD , Bartlett JR , Feller JA , Bergman NR . Relationship between foot function and medial knee joint loading in people with medial compartment knee osteoarthritis. J Foot Ankle Res 2013;6:33. 23927830 10.1186/1757-1146-6-33 PMC3750767

[9] Rutherford DJ , Hubley‐Kozey CL , Deluzio KJ , Stanish WD , Dunbar M . Foot progression angle and the knee adduction moment: a cross‐sectional investigation in knee osteoarthritis. Osteoarthritis Cartilage 2008;16:883–9. 18182310 10.1016/j.joca.2007.11.012

[10] Kerrigan DC , Todd MK , Riley PO . Knee osteoarthritis and high‐heeled shoes. Lancet 1998;351:1399–401. 9593411 10.1016/S0140-6736(97)11281-8

[11] Shakoor N , Sengupta M , Foucher KC , Wimmer MA , Fogg LF , Block JA . Effects of common footwear on joint loading in osteoarthritis of the knee. Arthritis Care Res 2010;62:917–23. 10.1002/acr.20165 PMC2940270 20191571

[12] Simonsen EB , Svendsen MB , Norreslet A , Baldvinsson HK , Heilskov‐Hansen T , Larsen PK , et al. Walking on high heels changes muscle activity and the dynamics of human walking significantly. J Appl Biomech 2012;28:20–8. 22431211 10.1123/jab.28.1.20

[13] Barkema DD , Derrick TR , Martin PE . Heel height affects lower extremity frontal plane joint moments during walking. Gait Posture 2012;35:483–8. 22169388 10.1016/j.gaitpost.2011.11.013

[14] Dawson J , Thorogood M , Marks SA , Juszczak E , Dodd C , Lavis G , et al. The prevalence of foot problems in older women: a cause for concern. J Public Health Med 2002;24:77–84. 12141589 10.1093/pubmed/24.2.77

[15] Kerrigan DC , Johansson JL , Bryant MG , Boxer JA , Della Croce U , Riley PO . Moderate‐heeled shoes and knee joint torques relevant to the development and progression of knee osteoarthritis. Arch Phys Med Rehabil 2005;86:871–5. 15895330 10.1016/j.apmr.2004.09.018

[16] Sherrington C , Menz HB . An evaluation of footwear worn at the time of fall‐related hip fracture. Age Ageing 2003;32:310–4. 12720618 10.1093/ageing/32.3.310

[17] Menz HB , Morris ME , Lord SR . Footwear characteristics and risk of indoor and outdoor falls in older people. Gerontology 2006;52:174–80. 16645298 10.1159/000091827

[18] Menant JC , Steele JR , Menz HB , Munro BJ , Lord SR . Optimizing footwear for older people at risk of falls. J Rehabil Res Dev 2008;45:1167–81. 19235118

[19] Menant JC , Steele JR , Menz HB , Munro BJ , Lord SR . Effects of footwear features on balance and stepping in older people. Gerontology 2008;54:18–23. 18253023 10.1159/000115850

[20] Murley GS , Landorf KB , Menz HB , Bird AR . Effect of foot posture, foot orthoses and footwear on lower limb muscle activity during walking and running: a systematic review. Gait Posture 2009;29:172–87. 18922696 10.1016/j.gaitpost.2008.08.015

[21] Paiva de Castro A , Rebelatto JR , Aurichio TR . The relationship between foot pain, anthropometric variables and footwear among older people. Appl Ergon 2010;41:93–7. 19497557 10.1016/j.apergo.2009.05.002

[22] Dufour AB , Broe KE , Nguyen US , Gagnon DR , Hillstrom HJ , Walker AH , et al. Foot pain: is current or past shoewear a factor? [Cross‐sectional study/cohort study]. Arthritis Rheum 2009;61:1352–8. 19790125 10.1002/art.24733 PMC2761974

[23] Fu K , Metcalf BR , Bennell KL , Zhang Y , Gross KD , Mills K , et al. Is heel height associated with pain exacerbations in hip osteoarthritis patients?‐Results from a case‐crossover study. J Clin Med 2020;9:1872. 10.3390/jcm9061872 PMC7356907 32560086

[24] Hart DJ , Mootoosamy I , Doyle DV , Spector TD . The relationship between osteoarthritis and osteoporosis in the general population: the Chingford Study. Ann Rheum Dis 1994;53:158–62. 8154931 10.1136/ard.53.3.158 PMC1005278

[25] McQueen P , Gates L , Marshall M , Doherty M , Arden N , Bowen C . The effect of variation in interpretation of the La Trobe radiographic foot atlas on the prevalence of foot osteoarthritis in older women: the Chingford general population cohort. J Foot Ankle Res 2017;10:54. 29234466 10.1186/s13047-017-0239-9 PMC5723087

[26] Kellgren JH , Lawrence JS . Radiological assessment of osteo‐arthrosis. Ann Rheum Dis 1957;16:494–502. 13498604 10.1136/ard.16.4.494 PMC1006995

[27] Felson DT , Naimark A , Anderson J , Kazis L , Castelli W , Meenan RF . The prevalence of knee osteoarthritis in the elderly: the Framingham Osteoarthritis Study. Arthritis Rheum 1987;30:914–8. 3632732 10.1002/art.1780300811

[28] Leyland KM , Gates LS , Nevitt M , Felson D , Bierma‐Zeinstra SM , Conaghan PG , et al. Harmonising measures of knee and hip osteoarthritis in population‐based cohort studies: an international study. Osteoarthritis Cartilage 2018;26:872–9. 29426005 10.1016/j.joca.2018.01.024 PMC6010158

[29] Perry TA , Wang X , Gates L , Parsons CM , Sanchez‐Santos MT , Garriga C , et al. Occupation and risk of knee osteoarthritis and knee replacement: a longitudinal, multiple‐cohort study. Semin Arthritis Rheum 2020;50:1006–14. 33007601 10.1016/j.semarthrit.2020.08.003 PMC9546524

[30] Felson DT , Niu J , Quinn EK , Neogi T , Lewis CL , Lewis CE , et al. Multiple nonspecific sites of joint pain outside the knees develop in persons with knee pain. Arthritis Rheumatol 2017;69:335–42. 27589036 10.1002/art.39848 PMC5292971

[31] Nguyen LY , Harris KD , Morelli KM , Tsai L‐C . Increased knee flexion and varus moments during gait with high‐heeled shoes: a systematic review and meta‐analysis. Gait Posture 2021;85:117–25. 33548909 10.1016/j.gaitpost.2021.01.017

[32] Dawson J , Juszczak E , Thorogood M , Marks SA , Dodd C , Fitzpatrick R . An investigation of risk factors for symptomatic osteoarthritis of the knee in women using a life course approach. J Epidemiol Community Health 2003;57:823–30. 14573590 10.1136/jech.57.10.823 PMC1732289

[33] McWilliams DF , Muthuri S , Muir KR , Maciewicz RA , Zhang W , Doherty M . Self‐reported adult footwear and the risks of lower limb osteoarthritis: the GOAL case control study. BMC Musculoskelet Disord 2014;15:308. 25240981 10.1186/1471-2474-15-308 PMC4190490

[34] Beckenkamp PR , Lin C‐WC , Chagpar S , Herbert RD , van der Ploeg HP , Moseley AM . Prognosis of physical function following ankle fracture: a systematic review with meta‐analysis. J Orthop Sports Phys Ther 2014;44:841–51. 25269609 10.2519/jospt.2014.5199

[35] Chun S‐W , Lim C‐Y , Kim K , Hwang J , Chung SG . The relationships between low back pain and lumbar lordosis: a systematic review and meta‐analysis. Spine J 2017;17:1180–91. 28476690 10.1016/j.spinee.2017.04.034

[36] Baaklini E , Angst M , Schellenberg F , Hitz M , Schmid S , Tal A , et al. High‐heeled walking decreases lumbar lordosis. Gait Posture 2017;55:12–4. 28407504 10.1016/j.gaitpost.2017.03.035

[37] Russell BS , Muhlenkamp KA , Hoiriis KT , Desimone CM . Measurement of lumbar lordosis in static standing posture with and without high‐heeled shoes. J Chiropr Med 2012;11:145–53. 23449540 10.1016/j.jcm.2012.02.002 PMC3437344

[38] Schroeder J , Hollander K . Effects of high‐heeled footwear on static and dynamic pelvis position and lumbar lordosis in experienced younger and middle‐aged women. Gait Posture 2018;59:53–7. 28987767 10.1016/j.gaitpost.2017.09.034

[39] Barton CJ , Coyle JA , Tinley P . The effect of heel lifts on trunk muscle activation during gait: a study of young healthy females. J Electromyogr Kinesiol 2009;19:598–606. 18472278 10.1016/j.jelekin.2008.03.001

[40] Murray MP , Mollinger LA , Gardner GM , Sepic SB . Kinematic and EMG patterns during slow, free, and fast walking. J Orthop Res 1984;2:272–80. 6491818 10.1002/jor.1100020309

[41] Mika A , Oleksy L , Mika P , Marchewka A , Clark BC . The effect of walking in high‐ and low‐heeled shoes on erector spinae activity and pelvis kinematics during gait. Am J Phys Med Rehabil 2012;91:425–34. 22311060 10.1097/PHM.0b013e3182465e57

[42] Salzman B . Gait and balance disorders in older adults. Am Fam Physician 2010;82:61–8. 20590073

[43] Yadollahpour N , Zahednejad S , Yazdi MJS , Esfandiarpour F . Clustering of patients with chronic low back pain in terms of physical and psychological factors: a cross‐sectional study based on the STarT Back Screening Tool. J Back Musculoskelet Rehabil 2020;33:581–7. 31658040 10.3233/BMR-181484

[44] Pincus T , Burton AK , Vogel S , Field AP . A systematic review of psychological factors as predictors of chronicity/disability in prospective cohorts of low back pain. Spine 2002;27:E109–20. 11880847 10.1097/00007632-200203010-00017

