# Supplementary material for: Effect of Heeled Shoes on Joint Symptoms and Knee Osteoarthritis in Older Adults: A 5‐Year Follow‐Up Study
Source: ACR Open Rheumatol. 2021 Jul 20;3(9):614–21. doi: 10.1002/acr2.11298 (PMC8449034; doi:10.1002/acr2.11298)
Supplement: Supplementary file 1 — Table S1 [file ACR2-3-614-s001.docx]

| Supplementary Table 1:  *Categorisation of job title to level of occupational loading; data from the Chingford Study.* | |
| --- | --- |
| Job Category | Level of occupational loading |
| Housewife | Light Manual |
| Higher Manager / professional | Sedentary (reference group) |
| Skilled Manager / teacher / nurse | Light Manual |
| Admin / secretary / clerical | Sedentary (reference group) |
| Skilled Manual | Heavy Manual |
| Unskilled Manual | Heavy Manual |
| Cleaning | Light Manual |
